# Periprocedural Myocardial Infarction: Do We Need an Updated Definition?

**DOI:** 10.3390/jcdd13030112

**Published:** 2026-03-02

**Authors:** Marcello Casuso Alvarez, Leonardo Luca Bavuso, Michele Di Leo, Marco Basile, Nicolò Vasumini, Tommaso Manaresi, Angelo Maida, Marco Moretti, Daniele Cavallo, Lisa Canton, Sara Amicone, Damiano Fedele, Elisa Conficoni, Alessandro Marinelli, Roberto Carletti, Francesco Angeli, Luca Bergamaschi, Matteo Armillotta, Carmine Pizzi

**Affiliations:** 1 Department of Medical and Surgical Sciences (DIMEC), Alma Mater Studiorum, University of Bologna, 40126 Bologna, Italy; marcello.casuso@studio.unibo.it (M.C.A.); leonardoluca.bavuso@studio.unibo.it (L.L.B.); michele.dileo@studio.unibo.it (M.D.L.); marco.basile4@studio.unibo.it (M.B.); nicolo.vasumini@studio.unibo.it (N.V.); tommaso.manaresi@studio.unibo.it (T.M.); angelo.maida@studio.unibo.it (A.M.); marco.moretti27@studio.unibo.it (M.M.); daniele.cavallo2@studio.unibo.it (D.C.); lisa.canton@studio.unibo.it (L.C.); sara.amicone@studio.unibo.it (S.A.); dmn.fed@gmail.com (D.F.); francesco.angeli1992@gmail.com (F.A.); luca.bergamaschi3@unibo.it (L.B.); matteo.armillotta3@unibo.it (M.A.); 2 Cardiology Unit, IRCCS Azienda Ospedaliero-Universitaria di Bologna, 40138 Bologna, Italy; 3 Cardiovascular Division, Morgagni-Pierantoni University Hospital, 47121 Forlì, Italy; elisa.conficoni@auslromagna.it (E.C.); alessandro.marinelli@auslromagna.it (A.M.); roberto.carletti@auslromagna.it (R.C.)

**Keywords:** periprocedural myocardial infarction, type 4a myocardial infarction, periprocedural myocardial injury, percutaneous coronary intervention, high-sensitivity cardiac troponin, Universal Definition of Myocardial Infarction

## Abstract

Periprocedural myocardial infarction after percutaneous coronary intervention (PCI) remains a debated entity, especially in the era of high-sensitivity cardiac troponin assays, which frequently detect biomarker rises even when clinically meaningful ischemia is absent. This review critically examines the main contemporary frameworks used to define these events, including the Fourth Universal Definition of Myocardial Infarction (UDMI), the Academic Research Consortium (ARC)-2 consensus, and the Society for Cardiovascular Angiography and Interventions (SCAI) definition, comparing biomarker thresholds, requirements for objective evidence of ischemia, and procedural criteria. We discuss how differences among definitions shape reported event rates and contribute to heterogeneity in event adjudication across studies. Key pathophysiologic mechanisms of myocardial injury during PCI are summarized, including side-branch compromise, distal embolization, microvascular dysfunction, and mechanical complications. Particular attention is given to the limitations of current criteria, such as incomplete assay standardization, variability in sampling timing, inconsistent reliability of ancillary criteria, including electrocardiography and imaging, and an uneven relationship between biomarker elevation and subsequent outcomes. Finally, we outline priorities for future updates, including harmonization of biomarker thresholds, greater emphasis on relative biomarker dynamics, and structured adjudication that integrates biomarkers with objective ischemic evidence. These steps may improve diagnostic specificity, reduce misclassification, and strengthen the clinical and trial relevance of periprocedural ischemic endpoints.

## 1. Introduction

Myocardial infarction (MI) remains a leading cause of mortality and morbidity worldwide [1]. Its clinical presentation reflects heterogeneous pathophysiological mechanisms, and diagnosis is currently framed by the Fourth Universal Definition of Myocardial Infarction (UDMI) [2]. According to the UDMI, MI requires evidence of acute myocardial injury, typically a rise and/or fall of cardiac troponin (cTn) with at least one value above the 99th percentile upper reference limit, together with evidence of acute myocardial ischemia derived from clinical presentation, electrocardiography, imaging, or angiography [3]. In contemporary practice, percutaneous coronary intervention (PCI) represents the most frequently adopted revascularization strategy in patients presenting with MI, particularly when rapid reperfusion is indicated [4]. Over time, progressive advances in interventional cardiology techniques and pharmacological therapy for ischemic heart disease have contributed to a substantial reduction in PCI-related complications [5]. Nonetheless, postprocedural elevations of myocardial necrosis biomarkers remain common in routine clinical practice, particularly after the widespread introduction of high sensitivity cardiac troponin (hs-cTn) assays [6,7]. In this setting, an increase in cTn may reflect periprocedural myocardial injury or, when the underlying mechanism is ischemic, periprocedural MI, classified as type 4a MI by the UDMI [3]. Beyond the UDMI, several scientific societies have proposed complementary, procedure focused definitions, most notably those from the Academic Research Consortium-2 (ARC-2) and the Society for Cardiovascular Angiography and Interventions (SCAI), which are primarily intended to standardize endpoints in the context of coronary revascularization and clinical trials and generally use the UDMI as a reference [8,9]. These definitions are not designed to be applied in isolation, nor are they mutually exclusive. When interpreted with clinical judgment, they can be integrated to better contextualize postprocedural myocardial injury and infarction across diverse settings. Importantly, they differ in diagnostic thresholds, required ancillary criteria, and the balance between sensitivity and prognostic specificity, which can translate into variability in event adjudication and clinical interpretation. Against this background, the purpose of the present review is to critically evaluate current definitions of periprocedural MI and discuss whether revisions may be warranted in light of evolving evidence and the steadily increasing volume of PCI performed in both acute and chronic clinical contexts.

## 2. Current Definitions of Periprocedural MI

### 2.1. Diagnostic Frameworks and Criteria

Periprocedural MI remains an area of ongoing debate, and a universally accepted definition has not been established [6]. The three main reference frameworks currently used to define periprocedural MI are UDMI, ARC-2, and SCAI [3,8,9]. A major source of heterogeneity across these frameworks is the distinction between periprocedural myocardial injury and periprocedural MI. This distinction reflects both the magnitude and dynamics of biomarker changes and, in some definitions, the requirement for objective evidence of new myocardial ischemia. The Fourth UDMI defines periprocedural myocardial injury as any post-PCI increase in cTn above the 99th percentile upper reference limit (URL) in patients with normal baseline values. In the same setting, periprocedural MI requires a higher biomarker threshold, namely a cTn increase to more than five times the 99th percentile URL, together with evidence of new onset myocardial ischemia. This may be documented by new ischemic ECG changes or development of new pathological Q waves, new imaging findings consistent with ischemia, or procedure-related complications of revascularization consistent with an ischemic mechanism. In patients with pre-procedural cTn values already above the URL, when levels are stable (variation 20% or less) or falling, UDMI requires a post-PCI rise greater than 20% within the first 48 h, with at least one value exceeding five times the 99th percentile URL, and the presence of at least one additional ischemic criterion to adjudicate periprocedural MI [3]. ARC-2 and SCAI adopt more stringent biomarker thresholds, including cTn elevations greater than 35 times the URL, and differ in how ancillary criteria are incorporated [8,9]. Finally, within the SCAI framework, periprocedural MI may be adjudicated even in the absence of ancillary criteria documenting new onset ischemia when cTn increases exceed 70 times the URL (Table 1) [9].

### 2.2. Clinical Setting and Incidence Variability

Event adjudication for periprocedural ischemic events differs substantially across clinical settings. In elective or chronic scenarios, baseline cTn is often below the URL, or, if elevated, tends to be stable, which facilitates the attribution of a new postprocedural rise to PCI [6]. In acute scenarios, cTn is frequently already elevated above the URL. Therefore, periprocedural myocardial injury and MI can be adjudicated with greater confidence only when the pre-PCI values are stable or falling, allowing a new, clinically meaningful rise related to the procedure to be distinguished from the index event [3,10].

From an epidemiological perspective, reported incidence varies according to the definition applied and the cardiac biomarker used. Even when restricting event adjudication to UDMI, rates of periprocedural MI remain heterogeneous and differ by clinical setting [6]. This distinction is particularly relevant when comparing chronic elective PCI with acute coronary syndrome (ACS) populations. In elective or chronic scenarios, UDMI based periprocedural MI has been reported in approximately 2% to 20% of cases [11]. In acute scenarios, variability persists. In patients with non–ST-segment elevation acute coronary syndrome (NSTE-ACS), two recent studies using UDMI reported different rates, around 5% versus 17%, highlighting how baseline biomarker patterns, sampling timing, and procedural complexity can materially influence event classification [10,12]. Overall, this variability contributes to heterogeneity in reported incidence across registries and trials and affects the apparent prognostic signal associated with periprocedural events.

## 3. Pathophysiologic Mechanisms of Periprocedural MI

**Figure 1:**
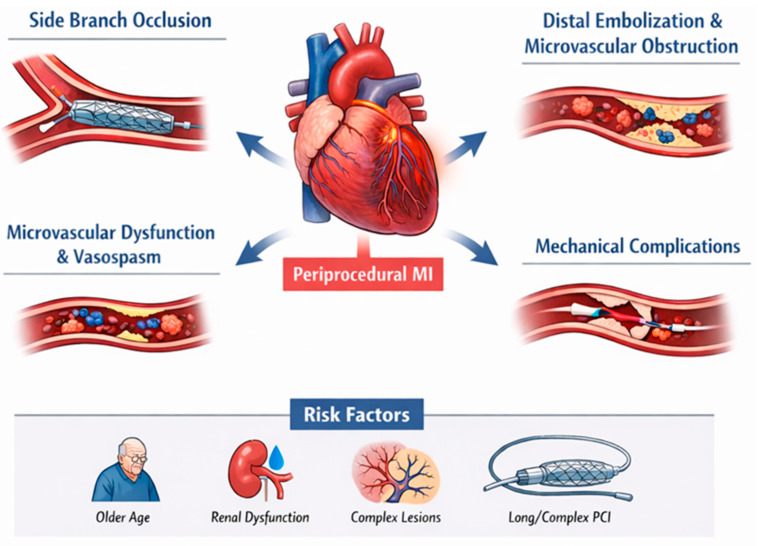
Pathophysiologic mechanisms of periprocedural myocardial infarction during PCI.

The etiology of periprocedural MI may be related to patient intrinsic vulnerability, PCI associated events, or procedural complications. These mechanisms frequently overlap and often coexist, reflecting a continuum of myocardial and microvascular injury during PCI (Figure 1) [6]. However, their relative contribution may differ between ACS and elective PCI settings.

### 3.1. Side Branch Occlusion

Side branch occlusion is among the most frequently documented angiographic mechanisms of periprocedural MI. In bifurcation PCI, stent deployment in the main vessel can reduce side branch ostial area and limit flow to downstream myocardium [13]. Intravascular imaging studies suggest that carina shift associated with main vessel expansion often contributes more than plaque shift, and excessive distal overexpansion may increase the likelihood of clinically relevant side branch compromise [14]. Large angiographic series and pooled data have reported side branch occlusion as a leading angiographic correlate of periprocedural MI, accounting for a substantial proportion of adjudicated events [15,16].

### 3.2. Distal Embolization and Microvascular Obstruction

Distal embolization of plaque or thrombotic material during PCI can result in slow flow or no reflow, with downstream microvascular obstruction. The reported frequency varies widely with the detection method, from angiography to more sensitive imaging approaches [17,18]. Intravascular imaging and spectroscopy have linked features of plaque vulnerability to periprocedural myonecrosis. In the CANARY trial, higher near-infrared spectroscopy lipid core burden was associated with an increased risk of post-PCI biomarker release, although embolization and myonecrosis can also occur in lesions without lipid rich features, and protection devices have not consistently reduced event rates in native vessels [19].

### 3.3. Microvascular Dysfunction and Vasomotor Disturbances

Microvascular dysfunction can persist despite restoration of epicardial patency [20,21,22]. Platelet activation with distal microthrombi, endothelial dysfunction, and the release of vasoconstrictor mediators may contribute to impaired myocardial perfusion after PCI. In this context, coronary vasospasm, including microvascular spasm and spasm distal to the treated segment, may act as a contributory mechanism and can present clinically as slow flow or no reflow [23]. Overall, no reflow should be viewed as the net result of embolic burden, microthrombi, endothelial dysfunction, and vasomotor abnormalities rather than a single dominant mechanism [6,13].

### 3.4. Mechanical Complications and Other Causes

Less frequent but high impact mechanisms include dissection, abrupt closure, acute stent thrombosis, and perforation [6]. Coronary artery perforation is rare but associated with adverse outcomes, and risk increases with older age, female sex, chronic kidney disease (CKD), chronic total occlusion (CTO) PCI, saphenous vein graft intervention, rotational atherectomy, and other complex techniques [24]. Additional uncommon contributors include wire-related injury, air embolism, arrhythmias during balloon inflations, and transient vessel occlusion, which may still be associated with post-PCI troponin rises [25].

### 3.5. Risk Determinants and Clinical Relevance

Patient factors such as older age, renal dysfunction, and elevated baseline troponin, together with lesion complexity including multivessel disease, left main disease, and bifurcation anatomy, have been associated with higher rates of periprocedural MI after PCI [26,27,28]. Procedural predictors include longer stent length, multivessel PCI, and technically complex interventions such as CTO strategies and the use of atherectomy techniques [10,15,16,29]. In addition, pre-procedural antiplatelet strategies may modulate the overall periprocedural risk profile, although evidence on routine P2Y_12_ receptor inhibitor pretreatment remains conflicting. While pretreatment in ST-segment elevation MI has been associated with improved cardiovascular outcomes [30], in patients with non–ST-segment elevation MI or chronic coronary syndrome (CCS) it has not shown clear benefits in reducing periprocedural MI and may increase in-hospital bleeding and delay surgical revascularization when CABG is indicated [1]. This appears consistent across pretreatment with clopidogrel [31,32,33], as well as with more potent agents such as ticagrelor and prasugrel [34,35], which have not demonstrated advantages over clopidogrel in the setting of elective PCI.

The etiologic distinction is clinically relevant because events linked to angiographic complications tend to show larger biomarker rises and worse prognosis than biomarker only elevations without a clear mechanical correlate [15,16]. This has practical implications for management, since angiographically evident complications typically require immediate corrective strategies, whereas isolated biomarker rises more often prompt closer monitoring and the optimization of secondary prevention [36]. Mechanism oriented prevention follows a similar logic, including strategies to limit side branch occlusion in high risk bifurcations and approaches aimed at reducing embolic burden and treating no reflow when it occurs [37].

## 4. Limitations and Pitfalls of Current Definitions

### 4.1. Diagnostic Challenges and Biomarker Sensitivity

One of the main diagnostic challenges for periprocedural MI is the widespread adoption of hs-cTn assays, which detect even minimal degrees of myocyte necrosis and make post-PCI biomarker increases very common [38]. As a result, separating clinically meaningful periprocedural MI from myocardial injury is often difficult. Several studies suggest that small to moderate post-PCI cTn elevations, especially when isolated, may not translate into worse long-term outcomes, indicating that low biomarker thresholds may be overly sensitive and may overclassify events with limited prognostic relevance [39,40]. This has prompted proposals to adopt higher cut-offs and to incorporate biomarker dynamics, including larger relative changes from baseline, to improve specificity and reduce false positive adjudication [10,26]. A second limitation is the inconsistent use and variable performance of ancillary ischemic criteria. Post-PCI ECG changes may be transient or non-specific, echocardiography may miss small infarcts, and more sensitive modalities such as cardiac magnetic resonance are not widely available for routine assessment [41,42,43,44,45]. When biomarker thresholds are combined with ECG or imaging abnormalities, prognostic correlations appear stronger, supporting a multimodal approach rather than reliance on biomarkers alone [46,47]. Attribution is further complicated by overlap with non-ischemic causes of cTn elevation. In severe CKD, hs-cTn may be chronically elevated due to structural myocardial changes and other factors, making the interpretation of post-PCI changes dependent on reliable baseline assessment and a careful evaluation of relative variation, while recognizing that ischemic and non-ischemic mechanisms may coexist periprocedurally [48,49].

### 4.2. Prognostic Implications and Study Heterogeneity

These diagnostic limitations translate into prognostic uncertainty and heterogeneity across studies. Indeed, several studies have provided contrasting results on the association between periprocedural MI and major adverse cardiovascular events (MACE) [50]. From these inconsistencies, a considerable number of meta-analyses of studies, mostly from the chronic/elective PCI setting, have confirmed the association between post-PCI cTn elevation and the risk of MACE. The most recent meta-analysis suggested that UDMI identifies more events, consistent with higher sensitivity but lower specificity for outcomes, whereas SCAI, which applies higher diagnostic thresholds for cTn, identifies fewer events with stronger associations with adverse clinical outcomes, and ARC-2 shows an intermediate performance [11].

Evidence on the prognostic role of periprocedural myocardial injury and infarction in selected ACS populations has long been limited, since few studies have enrolled ACS patients, who were in any case underrepresented compared with elective PCI cohorts. Recently, two studies demonstrated that periprocedural MI, as defined by the Fourth UDMI, is associated with a significant increased risk of all-cause mortality at 1 year in patients with NSTE-ACS [10,12].

However, it remains to be clarified whether the adverse prognostic role of periprocedural MI is primarily attributable to its causal role or whether it merely serves as a marker of baseline risk, atherosclerosis burden, procedural complexity, and subsequent adverse events [51].

### 4.3. Prognostic Implications of Biomarker Dynamics and Clinical Criteria

Building on the challenges related to the definition of periprocedural myocardial injury and infarction, it is essential to examine how the interaction between biomarker dynamics and clinical criteria influences prognostic outcomes across different clinical settings [52]. In particular, the interpretation of biomarker dynamics differs substantially between ACS and CCS. In elective PCI for CCS, Ueki et al. reported that ancillary ischemic criteria added prognostic information for UDMI periprocedural MI, but that their incremental value became less evident when higher biomarker thresholds were used in ARC-2 and SCAI definitions, suggesting that the relative contribution of non-biomarker criteria depends on the biomarker cut-off applied [5]. Conversely, in patients with NSTE-ACS, Leonardi et al. recently reported that isolated cTn increases, even when meeting very high thresholds, were not associated with higher mortality risk in the absence of ancillary criteria [12], perhaps because, in this setting, the absolute cTn values reached after PCI are more influenced by the pre-PCI values of the index infarct, thus reducing their prognostic relevance.

More broadly, cTn is a continuous variable, and thresholds differ across studies according to the biomarker used, the metric analyzed (absolute peak, fold URL, relative change), and the clinical setting, with limited prospective validation [53,54]. The 2021 European Society of Cardiology (ESC) Working Group on Cellular Biology of the Heart and European Association of Percutaneous Cardiovascular Interventions (EAPCI) consensus defined major periprocedural myocardial injury after elective PCI in CCS as a peak cTn more than five times the URL in patients with normal baseline values, based on pooled analyses suggesting prognostic relevance across assays [6,26]. Other cohorts proposed alternative thresholds, such as hs-cTn peaks more than eight times the URL predicting long-term adverse events in CCS with normal baseline values [55], while Ueki et al. did not observe an association between ESC/EAPCI defined major injury and 1-year cardiac mortality in a population with frequent baseline troponin elevation [5]. The optimal biomarker also remains debated. In elective left main PCI with largely normal baseline biomarkers, creatine kinase-MB (CK-MB) peaked more than three times the URL predicted long-term outcomes, whereas no troponin threshold independently predicted prognosis, although generalizability may be limited in contemporary practice where hs-cTn is standard [1,56]. In acute presentations, Armillotta et al. reported that periprocedural myocardial injury in non-ST segment elevation MI with stable or decreasing baseline cTn was associated with 1-year mortality and adverse events only when post-PCI cTn increased by more than 40% relative to the baseline, whereas smaller increases were not prognostically informative [10].

Overall, across both chronic and acute settings, the prognostic signal of post-PCI biomarker release appears to depend on the magnitude and pattern of change and, importantly, on the concomitant presence of objective ischemic or procedural correlates. This supports approaches that integrate biomarker dynamics with ancillary criteria rather than relying on a single universal cut-off [6]. Prospective validation is still needed to identify thresholds and combinations of criteria that maximize clinical relevance and reproducibility across different PCI populations.

### 4.4. Management Implications

Currently, there is no scientific evidence on the optimal management of patients diagnosed with periprocedural MI. International guidelines for the management of chronic and acute coronary syndromes do not provide specific recommendations for this condition [1,57,58,59]. The 2021 ESC/EAPCI consensus paper suggests that patients with ST-segment elevation following PCI should immediately undergo angiographic re-evaluation. It also recommends that patients with “major” periprocedural myocardial injury (as defined by Silvain et al., [26]) or MI should be treated to minimize the risk of future MACE [6]. However, it remains unclear whether therapies typically used for spontaneous MI—such as beta-blockers, angiotensin-converting enzyme inhibitors, or intensive lipid-lowering therapy targeting lower low-density lipoprotein (LDL) cholesterol levels—are beneficial for patients with periprocedural MI in the absence of other clinical indications [6]. Consequently, the management of these patients is currently individualized, relying heavily on clinical judgment.

## 5. Toward More Robust Definitions of Periprocedural Ischemic Events

**Figure 2:**
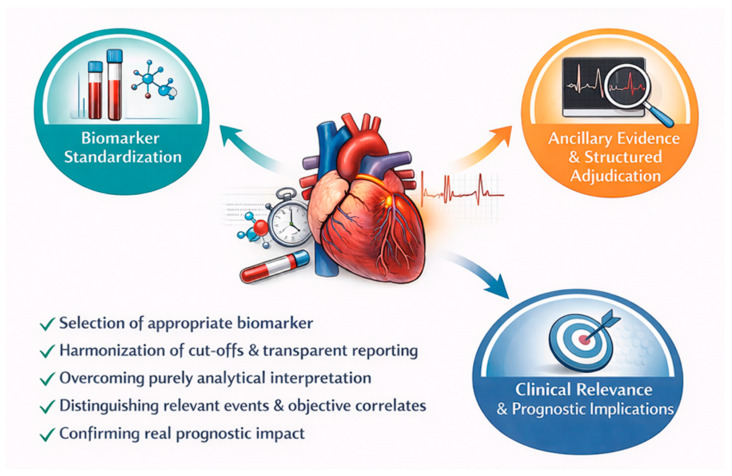
From biomarkers to clinical relevance: redefining periprocedural ischemic events.

A future UDMI update will require global consensus and prospective validation to ensure that the revised criteria are clinically meaningful and reproducible across different PCI settings and patient populations. Key priorities include the selection of the appropriate biomarker to be dosed, harmonizing biomarker-based thresholds, and strengthening structured adjudication that integrates biomarker dynamics with objective evidence of ischemia (Figure 2).

### 5.1. Biomarker Standardization and Assay Transparency

A consistent diagnostic framework requires explicit standardization of the biomarker strategy. Similar event rates may be observed with conventional assays and hs-cTn, but this does not imply analytical equivalence, because sensitivity, precision, and the ability to capture dynamic changes differ and directly influence both relative variations and absolute cut-offs [60,61,62]. Combining results derived from different assay types increases methodological heterogeneity, complicates endpoint adjudication, and undermines comparability across centers and over time [61,63,64,65]. Given that hs-cTn is the predominant biomarker in contemporary practice [1,66], future criteria based on relative changes and multiple absolute thresholds should be validated primarily in hs-cTn-based settings and should not be extrapolated to conventional assays or CK-MB without dedicated evidence. Clear reporting of assay type, URL methodology, and sampling timing should be considered essential to ensure consistent diagnosis and reproducible research endpoints. Finally, adopting relative changes in troponin levels instead of fold increases relative to the upper reference limit would simplify patient management, especially for those with elevated baseline troponin levels (common in ACS populations), and reduce discrepancies arising from the use of different assays [10].

### 5.2. Strengthening Ancillary Evidence and Structured Adjudication

A major opportunity for improving current definitions is more systematic acquisition and interpretation of ancillary evidence of ischemia and procedure-related complications. In biomarker-driven frameworks, particularly in NSTE-ACS where the baseline cTn is often elevated, failure to document ancillary criteria increases the risk of misclassifying analytically driven biomarker variability as clinically meaningful events [12]. A structured adjudication approach that explicitly prompts the assessment of ECG, angiographic findings, and imaging evidence would improve specificity and better align event classification with mechanisms that are actionable during or immediately after PCI [6,52]. Standardized reporting of procedural complications and predefined ischemic criteria would also reduce interobserver variability and facilitate more reliable comparisons across studies.

### 5.3. Clarifying What Constitutes Clinically Meaningful Periprocedural Myocardial Infarction

Accumulating data indicate that not all periprocedural biomarker rises carry prognostic significance [50]. Across both chronic and acute settings, modest relative increases may have limited association with mortality or MACE, whereas larger relative changes appear more consistently linked to adverse outcomes, supporting the concept that current criteria may not optimally identify clinically meaningful events [10,11]. This has important implications for both clinical care and research because an overly sensitive framework risks labeling a substantial number of patients with events that do not reflect clinically relevant myocardial ischemia [67]. Future definitions should therefore make the conceptual separation explicit by adopting a pragmatic working definition that aligns event classification with clinical relevance: a clinically meaningful periprocedural MI could be considered when a relevant hs-cTn rise exceeds a sufficiently high threshold to improve specificity, together with at least one objective ischemic/procedural correlate (e.g., angiographic flow-limiting complication, new ischemic ECG changes/pathological Q waves, or imaging evidence of new myocardial loss). This would preserve the conceptual distinction between biomarker-defined injury and periprocedural MI while prioritizing events most likely to reflect true ischemia and adverse prognosis. This would also reduce the variability introduced by subjective elements, including interobserver differences in ECG interpretation and imaging or angiographic assessment [68,69,70].

### 5.4. Enhancing Prognostic Implications in Trial Design

Another essential aspect of revising the definition of periprocedural MI is its impact on the design and interpretation of clinical trials [46]. Given that periprocedural MI does not always correlate well with long-term adverse outcomes, it makes it difficult to serve as a reliable surrogate for more serious endpoints, such as mortality [50,71,72]. Its use, either as a standalone endpoint or as part of composite endpoints, may limit the interpretability of trial results, especially when weighted equally with spontaneous MI, which has a well-established and more severe prognosis [73]. This issue has been highlighted in large randomized clinical trials comparing myocardial revascularization strategies, such as the ISCHEMIA trial and the EXCEL trial [74,75]. Post hoc analyses have shown that using different definitions of periprocedural MI can result in significant differences in endpoint incidence and potentially lead to divergent interpretations of study outcomes [76]. For future trial design, it is essential to establish a definition of myocardial injury and periprocedural MI supported by strong evidence, allowing for a weighted comparison of its prognostic impact with that of spontaneous MI [77,78]. Specifically, in trials investigating periprocedural MI, utilizing imaging endpoints, such as cardiac magnetic resonance, may provide quantitative data to further validate diagnostic criteria and troponin thresholds under review [41,79].

Future prospective studies should therefore apply standardized biomarker sampling and prespecified ancillary criteria with centralized adjudication, and test candidate thresholds/delta metrics across both elective PCI and ACS populations against patient-centered outcomes. Such designs would help identify definitions that are reproducible and clinically meaningful, and clarify the extent to which periprocedural events add prognostic information beyond baseline risk and procedural complexity.

## 6. Conclusions

Defining periprocedural MI remains a complex and evolving challenge, shaped by advancements in biomarkers, diagnostic criteria, and clinical practice. While the Fourth UDMI, along with frameworks like ARC-2 and SCAI, provides valuable diagnostic tools, variability in their application and the evolving understanding of biomarker dynamics have introduced significant diagnostic and prognostic uncertainty. The integration of biomarkers with clinical and procedural evidence—such as ECG changes, imaging findings, and complications during PCI—could enhance specificity, improve event adjudication, and reduce misclassification.

As PCI continues to grow in both acute and chronic settings, the need for a more robust and standardized definition becomes clearer. Future revisions to the UDMI should focus on harmonizing biomarker thresholds, incorporating dynamic changes in biomarker levels, and providing more precise guidelines for the integration of clinical criteria to better align clinical decisions with the true nature of periprocedural ischemic events. By strengthening the clinical relevance of these definitions, we can achieve more accurate risk stratification, leading to improved patient outcomes and more reliable endpoints in clinical trials. Ultimately, a more refined and context-specific approach will help balance sensitivity and specificity, ensuring that periprocedural MI is more reliably linked to adverse outcomes, thereby guiding more effective management strategies.

## Figures and Tables

**Table 1:** Diagnostic criteria for periprocedural myocardial injury and infarction after PCI in patients with normal baseline cardiac biomarkers: comparison of contemporary definitions.

Framework	Periprocedural Myocardial Injury	Periprocedural Myocardial Infarction	Additional Evidence Required
Fourth UDMI (2018) [3]	Any post-PCI cTn increase above the 99th percentile URL within 48 h	cTn > 5× URL within 48 h with ancillary criteria	At least one of the following: new ischemic ECG changes new pathological Q waves imaging evidence of new loss of viable myocardium or new regional wall motion abnormality consistent with ischemic etiology angiographic evidence of a PCI-related flow limiting complication postmortem evidence of procedure-related coronary thrombus or a large circumscribed area of necrosis
ARC-2 (2018) [8]	cTn ≥ 70× URL within 48 h	cTn ≥ 35× URL within 48 h with ancillary criteria	At least one of the following: new significant Q waves or equivalent flow limiting angiographic complication new substantial myocardial loss on imaging
SCAI (2013) [9]	Not defined as a separate category	CK-MB > 10× URL without ancillary criteria CK-MB > 5× URL with ancillary criteria cTn > 70× URL without ancillary criteria cTn > 35× URL with ancillary criteria	At least one of the following: new pathological Q waves in at least 2 contiguous leads new persistent LBBB

PCI, percutaneous coronary intervention; UDMI, Universal Definition of Myocardial Infarction; ARC-2, Academic Research Consortium-2; SCAI, Society for Cardiovascular Angiography and Interventions; cTn, cardiac troponin; URL, upper reference limit; h, hours; CK-MB, creatine kinase-myocardial band; ECG, electrocardiography; LBBB, left bundle branch block.

## Data Availability

No new data were created or analyzed in this study. Data sharing is not applicable to this article.

## Abbreviations

The following abbreviations are used in this manuscript:

ACS	Acute coronary syndrome
ARC-2	Academic Research Consortium 2
CABG	Coronary artery bypass grafting
CCS	Chronic coronary syndrome
CK-MB	Creatine kinase myocardial band
CKD	Chronic kidney disease
cTn	Cardiac troponin
CTO	Chronic total occlusion
ECG	Electrocardiography
EAPCI	European Association of Percutaneous Cardiovascular Interventions
ESC	European Society of Cardiology
hs-cTn	High-sensitivity cardiac troponin
LDL	Low-density lipoprotein
MACE	Major adverse cardiovascular events
MI	Myocardial infarction
NSTE-ACS	Non-ST-segment elevation acute coronary syndrome
PCI	Percutaneous coronary intervention
SCAI	Society for Cardiovascular Angiography and Interventions
UDMI	Universal Definition of Myocardial Infarction
URL	Upper reference limit
